# The NACA score predicts mortality in polytrauma patients before hospital admission: a registry-based study

**DOI:** 10.1186/s13049-024-01281-3

**Published:** 2024-11-18

**Authors:** Paolo Ivan Fiore, Andrea Stefano Monteleone, Jochen Müller, Giuseppe Filardo, Christian Candrian, Martin Riegger

**Affiliations:** 1 Service of Orthopaedics and Traumatology, Department of Surgery, EOC, Via Tesserete 46, 6900 Lugano, Switzerland; 2 Faculty of Biomedical Sciences, Università della Svizzera Italiana, Lugano, Switzerland

**Keywords:** Polytrauma, NACA score, ISS, GCS, Emergency medicine, Mortality

## Abstract

**Background:**

The early assessment of the severity of polytrauma patients is key for their optimal management. The aim of this study was to investigate the discriminative performance of the NACA score in a large dataset by stratifying the severity of polytraumatized patients in correlation to injury severity score (ISS), Glasgow Coma Scale (GCS), and mortality.

**Methods:**

This study on the Swiss Trauma Registry investigated 2239 polytraumatized patient (54.3 ± 22.8 years) enrolled from 2015 to 2023: 0.5% were NACA 3, 76.7% NACA 4, 21.4% NACA 5, and 1.4% NACA 6. The NACA predictive value of patients’ mortality was investigated, as well as the correlation of ISS and GCS scores, and other factors influencing patients’ survival at discharge and after 28 days.

**Results:**

In NACA 4 and 5 the survival rate during hospitalization was 97.7% and 82.5%, respectively, and 28-day mortality 3.5% and 23.5%, respectively (*p* < 0.0005). NACA correlated with GCS in the prehospital phase and in the emergency room (*p* < 0.0005), as well as with ISS (*p* < 0.0005). NACA 4 and 5 presented different injury patterns (fall < 3 m vs vehicle accident) with NACA 5 requiring more CPR and intubation (*p* < 0.001, *p* < 0.0005). The ROC AUC analysis showed the prehospital NACA and GCS values as the strongest variables predicting patients’ survival.

**Conclusions:**

This study provides valuable evidence supporting the effectiveness of the NACA score in assessing the severity of polytrauma patients in both the pre-ER and ER condition. Considering the statistical significant correlation with the GCS and with the ISS, NACA is a valid score for assessing polytrauma patients.

## Background

Polytrauma are a major public health concern due the possible residual disability leading to a patient societal dependency and a loss of productive life, as well as to significant costs [1, 2]. Polytraumatized patients present a high risk of death, which can occur in the emergency department and during hospitalization treatment, as well as directly at the scene of injury [3]. Therefore, it is key to properly manage these patients from the earliest moments, and numerous risk stratification scores have been devised to assess the severity of polytrauma cases [4, 5]. These tools assist clinicians in categorizing case severity and, the knowledge of these tools is crucial to avoid errors during diagnostic and therapeutic steps and to initiate prompt diagnosis and treatment [6, 7]. Moreover, these trauma scoring systems have been established to speed up the assessment and appropriate allocation of resources in polytraumatized patients [8].

The National Advisory Committee for Aeronautics (NACA) score, originally formulated in the 1960s to evaluate trauma patient severity 24 h post-hospital admission. The score was subsequently modified by Tryba et al. to be used also in the prehospital setting and to expand its scope and manage medical cases based on the clinical severity [9]. Adopted by prehospital emergency medical services (EMS), the NACA score presents advantages being simple while encompassing different clinical scenarios, avoiding the need for specific clinical or biological parameters [10, 11]. In fact, its application involves classifying patients based on the anticipated outcome of their existing injuries, and a recent studies demonstrated the effectiveness in predicting 48-h and 30-day mortality [10, 12]. However, despite its widespread use, data about the correlation between the NACA score and the overall mortality are still lacking, and the patient severity is heterogeneously documented, also relying to other scores like the Injury Severity Score (ISS) or the Glasgow Coma Scale (GCS) [4]. Accordingly, the study of a large dataset investigated with all three scores would provide clinically relevant indications on the potential of this score system to stratify patients based on trauma severity.

The primary outcome of this study was to investigate the discriminative performance of the NACA score in a large well-defined dataset by stratifying the severity of polytraumatized patients in correlation to parameters such as the ISS, the GCS, and mortality. As secondary outcome, the study aimed to explore variables that may affect patient survival within each category of the NACA score.

## Methods

### Study design

This study was conducted at the Lugano Regional Hospital, a Level I Trauma Center, after the approval of the local Ethical Committee (prot 2024-00236 CE 4536). Data was withdrawn from the local polytrauma registry affiliated to the National Swiss Trauma Registry (STR). This registry collects the comprehensive documentation of data from prehospital stages to the conclusion of treatment. Data are collected from the ambulance protocol to the discharge letter. Data from both the prehospital as well the hospital settings are collected prospectively. Each polytraumatized patient was included in this study from the registry’s introduction in 2015. Patient severity is assessed by the prehospital emergency-trained physician or paramedic at the accident location resulting in the NACA score [9]. The data collected included both prehospital, hospital, and the follow-up mortality within 28-days after hospital discharge, as well as ISS and GCS. Exclusion criteria were NACA 0–2, 7 and cases with missing data.

### Study population and data collection

Two authors (P.I.F. and A.S.M), reviewed the registry’s database retrieving patients of any age managed through the emergency department of the trauma center classified as polytrauma patients from January 2015 to December 2023 [13]. The following parameters were retrospectively collected: NACA score, age, mechanism of injury, GCS, resuscitation and intubation both in the prehospital and in the Emergency Room (ER), ISS, and death during the hospital stay or within 28-days after hospital discharge.

### Patient evaluation

### National advisory committee on aeronautics score (NACA)

The NACA score is a grading system consisting in a standardized scale used primarily in the pre-hospital emergency medical settings to assess the severity of the patient’s medical condition.

The score enables the emergency physicians or paramedics at the accident location to quickly and consistently assess the patients’ condition. It ranges from 0 to 7 based on the patient’s injury or disease and its medical needs [9]. Both physicians and paramedics are trained to evaluate patients’ severity, based on the vital signs and clinical status resulting in the NACA score with guidance provided through specific examples in order to ensure a more reliable scoring process. The NACA score is used at different time points in the care process: at the initial evaluation, during transport and upon hospital admission. It offers an immediate, standardized way to describe the severity of a patient’s condition, ensuring an efficient communication between the first responders’ physicians and the hospital. Table 1 illustrates the NACA score.

**Table 1:** The eight-level NACA* score extending from level 0 (no injury) to level 7 (death) [9]

NACA 0	No injury or disease
NACA 1	Injuries/diseases without any need for acute physicians’ care
NACA 2	Injuries/diseases requiring examination and therapy by a physician but hospital admission is not indicated
NACA 3	Injuries/diseases without acute threat to life but requiring hospital admission
NACA 4	Injuries/diseases which can possibly lead to deterioration of vital signs
NACA 5	Injuries/diseases with acute threat to life
NACA 6	Injuries/diseases transported after successful resuscitation of vital signs
NACA 7	Lethal injuries or diseases (with or without resuscitation attempt)

*NACA, National Advisory Committee on Aeronautics

### Injury severity score (ISS)

The injury severity score is an anatomical scoring system that provides an overall score ranging from 0 to 75 for polytrauma patients [4]. Each injury is assigned an Abbreviated Injury Scale (AIS) score and is allocated to one of six body regions. The highest AIS score in each body region is used. The three most severely injured body regions have their score squared and added together to produce the ISS score, so that an AIS score of 6 (unsurvivable injury) equalizes to an ISS score of 75. According to the STR and to the international community, an ISS ≥ 16 is considered a major trauma or polytrauma and these patients’ data are allocated in the STR [4, 14].

### Glasgow coma scale (GCS)

The GCS is used internationally to objectively describe the extent of impaired consciousness in all types of acute medical and trauma patients according to three aspects of responsiveness: eye-opening, motor, and verbal responses [15]. In the clinical practice it has been crucial to treat the patients’ clinical and neurological status according to the GCS since the GCS status can change in better or in worse between the emergency-trained physicians’ first assessment in the prehospital setting and during the primary survey at the ER. [8, 16, 17]

## Statistics

Descriptive statistics were performed to describe the sociodemographic and injury-related characteristics of samples. Continuous variables were expressed as pooled means with their confidence interval (CI) and standard deviation (SD). Qualitative variables were expressed as frequencies with their range. The Shapiro Wilk test was used to check Gaussian distribution of the quantitative variables, the Levene test was used to check homoscedasticity. The Spearman correlation was performed to assess the influence of the NACA score on quantitative variables. One Way Anova or the equivalent nonparametric test (either Kruskal Wallis or Mann Whitney) was utilized to assess NACA score differences among the groups. The Pearson’s chi-square test was performed to investigate relationships between dichotomous and grouping variables. The ROC Curve analysis was performed to assess the impact of quantitative variables on the survival. The Generalized Linear Model was used as multivariate analysis to assess the combined influence of the NACA score and the other collected variables on complications, length of hospitalization, and mortality.

## Results

The total number of polytraumatized patients admitted to the ER from 2015 to 2023 was 2239 with a mean age of 54.3 ± 22.8. Among the total number of patients, 0.5% were NACA 3, 76.7% were NACA 4, 21.4% were NACA 5, 1.4% were NACA 6. The detailed characteristics of the identified patients are described in Table 2.

**Table 2:** Patients’ characteristics

	NACA 3 (N = 11)	NACA 4 (N = 1718)	NACA 5 (N = 479)	NACA 6 (N = 31)
Age (mean ± SD)	56.3 ± 23.5	54.2 ± 23.0	54.9 ± 21.9	47.5 ± 22.2
Injury mechanism type (N, %)	4 other; 3 fall < 3 m; 1 pedestrian – road incident; 3 road traffic incident	262 other; 565 fall < 3 m; 281 fall > 3 m; 51 pedestrian – road incident; 559 road traffic incident	79 other; 123 fall < 3 m; 106 fall > 3 m; 23 pedestrian – road incident; 148 road traffic incident	3 other; 4 fall < 3 m; 8 fall > 3 m; 2 pedestrian – road incident; 14 road traffic incident
GCS prehospital (mean ± SD)	14.9 ± 0.3	14.2 ± 1.6	10.2 ± 4.6	3.8 ± 2.5
Reanimation prehospital (N, %)	0	1712 no (99.7%), 6 yes (0.4%)	470 no (98.1%), 9 yes (1.9%)	2 (6.5%) no, 29 (93.5%) yes
Intubation prehospital; at ER (N, %)	0; 1 (9.1%)	1700 (98.9%) no, 18 (1.1%) yes; 1651 (96.1%) 67 yes (3.9%)	272 (56.8%) no, 207 (43.2%) yes; 401 (83.7%) no, 78 (16.3%)	1 (3.2%) no, 30 (96.8%) yes; 28 (90.3) no, 3 (9.7) yes
GCS at ER (mean ± SD)	14.8 ± 0.4	14.3 ± 1.8	8.7 ± 5.5	3.0 ± 0
ICU stay (N, %)	1 (9.1%)	445 (25.9%)	342 (71.4%)	23 (74.2%)
Survival	100%	97.7%	82.5%	19.4%
ISS ≥ 16 (N, %)	2 (18.2%)	341 (19.8%)	264 (55.1%)	25 (80.6%)
Death < 28d after discharge (N, %)	6 yes, 5 unknown	49 yes, 1398 no, 271 unknown	84 yes, 357 no, 38 unknown	27 yes, 4 no

GCS: Glasgow Coma Scale; NACA score; ISS: Injury Severity Sore; ER: emergency room; ICU: intensive care unit; N: patients number; SD: standard deviation; d: days

### NACA score—predictive value of patients’ mortality

In the NACA 4 group there was a 97.7% survival rate during hospitalization and 3.5% mortality at 28 days of the discharged patients, while in the NACA 5 group there was a 82.5% survival rate during hospitalization and 23.5% mortality of the discharged patients at 28 days, respectively (there was some missing data in both groups on this aspect: 15.8% in NACA 4 and 7.9% in NACA 5). There was a correlation between NACA score and patients’ survival during the hospital stay and after 28 days after discharge (both *p* < 0.0005 Chi Squared Pearson Test). Therefore, NACA 4 predicts a statistically significant higher survival rate during the hospital stay as well as within one month after discharge than NACA 5 patients.

### Correlation NACA—GCS—ISS score

**Fig. 1:**
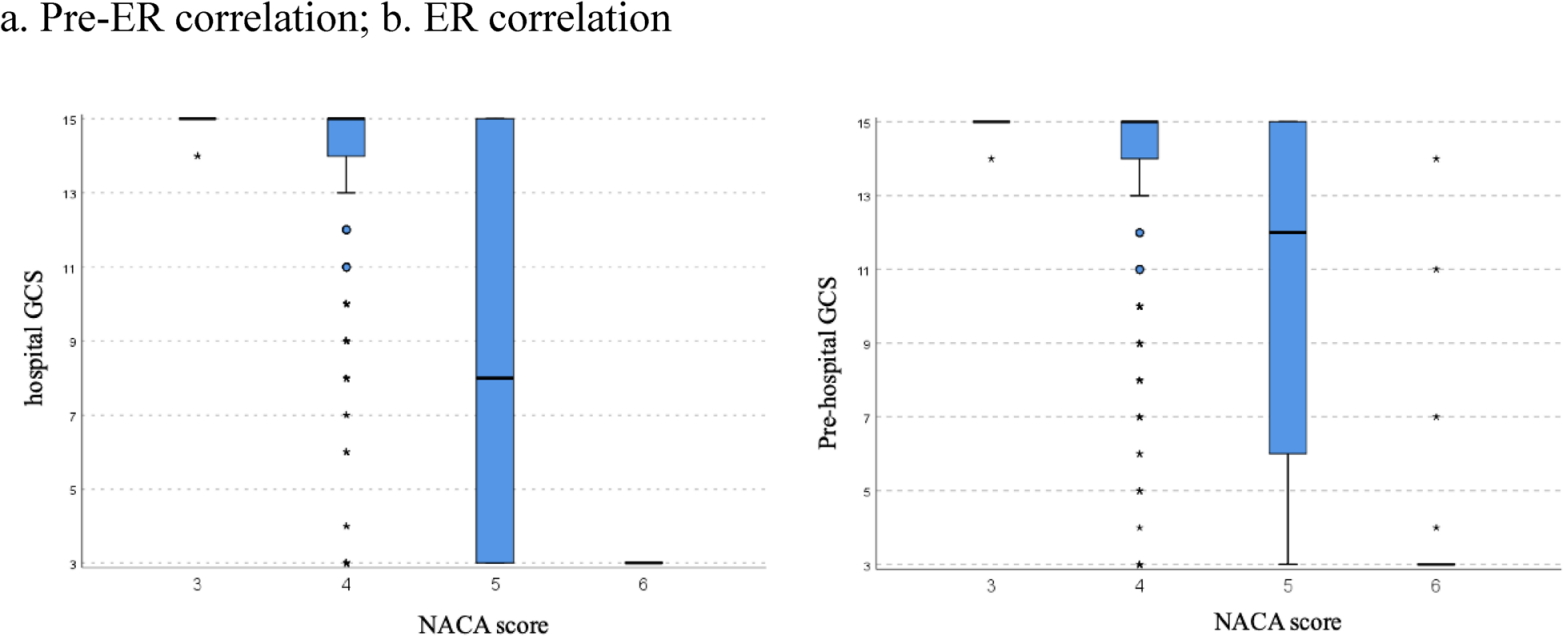
Correlation between the NACA and the GCS score. The black horizontal line shows the mean value, *represents the outliers. a. represents pre-ER correlation, b. ER correlation

The results of the NACA and GCS showed an inverse significant correlation both in the prehospital phase and in the ER (pre Kendall rank correlation coefficient = − 0.409, *p* < 0.0005; ER Kendall rank correlation coefficient = − 0.453, *p* < 0.0005), as shown in Fig. 1. The correlation NACA and ISS > 16 was statistically significant (*p* < 0.0005 Pearson's Chi Square test): A higher prehospital or ER-NACA score predicted a more likely ISS > 16 score (Table 3**).** Also, the Kendall rank correlation coefficient (0.302) showed a statistically significant correlation between the GCS and the ISS (*p* < 0.0005); this indicates the better the GCS status is at admission the lower is the ISS in general (25 missing ISS data not included in this analysis).

**Table 3:** Correlation NACA score and ISS

NACA		ISS < 16	ISS > 16	Total n	*p* value
3	n	10	1	11	*p* < 0.0005
	%	90.9	**9.1**	100.0	
4	n	1388	312	1700	
	%	81.6	**18.4**	100.0	
5	n	224	248	472	
	%	47.5	**52.5**	100.0	
6	n	5	25	30	
	%	16.7	**83.3**	100.0	
Total	n	1627	587	2214	
	%	73.5	26.5	100.0	

The number written in bold demonstrate that the higher the NACA score, the higher percentage of patients with an ISS >16 has been statistically found. As such the bold numbers represent this percentage that has been found statistically significant

**Fig. 2:**
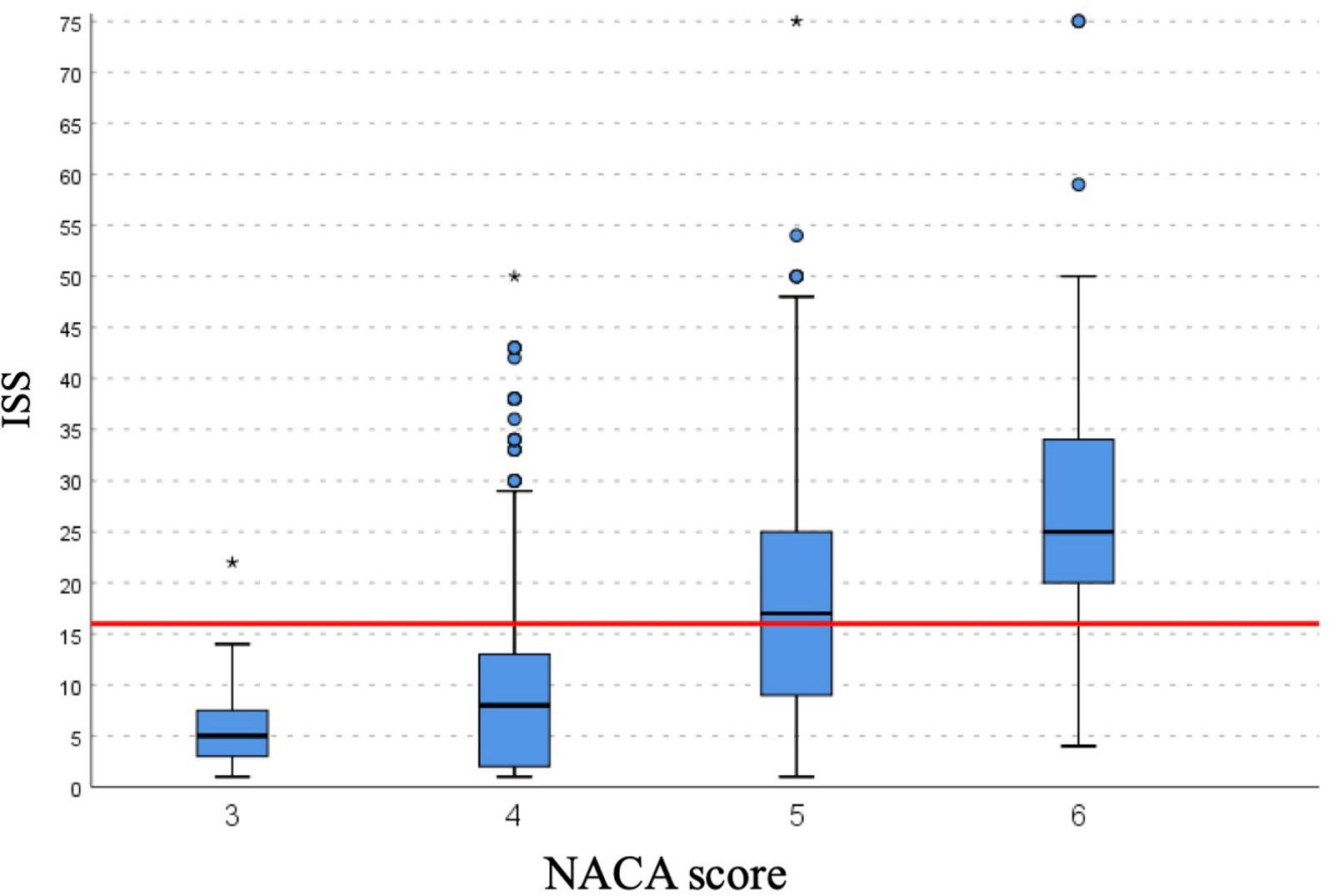
illustrates the cut-offs based on percentiles

NACA 3 and 4 had similar ISS values with a mean score below 16 (the minimum threshold for a polytrauma) whereas NACA 5 and 6 showed a mean ISS value of more than 16. The distinction between NACA 4 and 5 was a mean ISS value between 19 and 25, where 19 is in the 25th percentile of NACA 6 and 25 in the 75th percentile of NACA 5. The distinction between NACA 5 and 6 is a mean ISS value between 9 and 13, where 9 is actually part of the 25th percentile of the NACA 5 and 13 of the 75th percentile of the NACA 4 score (Fig. 2).

### Factors correlating with the NACA score

The different mechanisms of trauma were divided into 5 subcategories: fall from > 3 m (395 patients, 17.6%); fall from < 3 m (695 patients, 31.0%); pedestrian accidents (77 patients, 3.4%); road traffic accident (724 patients, 32.3%) with vehicle e.g. car, scooter, bike; and others (348 patients, 15.5%). A stratification based on the NACA score was done to investigate a correlation between the mechanism of trauma and the clinical severity of the patients. Due to the low number of cases in the NACA 3 and 6 groups, only NACA 4 and 5 groups were assessed statistically. In the NACA 4 group, the predominant mechanism of trauma was a fall < 3 m (32.9%), while in the NACA 5 group it was a vehicle accident (30.9%) which statistically different (*p* < 0.005 Chi Squared Pearson Test) (Table 2).

Further analysis was performed to investigate other factors correlating with the NACA score. Since “Reanimation (CPR) and intubation” of polytrauma patients in the prehospital management are important variables with a potential impact on patients’ survival, they were investigated for NACA 4 and 5, and a statistical difference was found between the NACA 4 and 5 group for both variables (CPR and pre-ER intubation) with a *p* value 0.001 and *p* < 0.0005 respectively. Since polytrauma patients of different age have different injury patterns with different survival rates during hospitalization and after discharge, age was investigated. However, the Kendall rank correlation coefficient showed no statistical difference between age and NACA score, neither with GCS and ISS.

### Receiver operating characteristic (ROC) analysis on patients’ survival

**Fig. 3:**
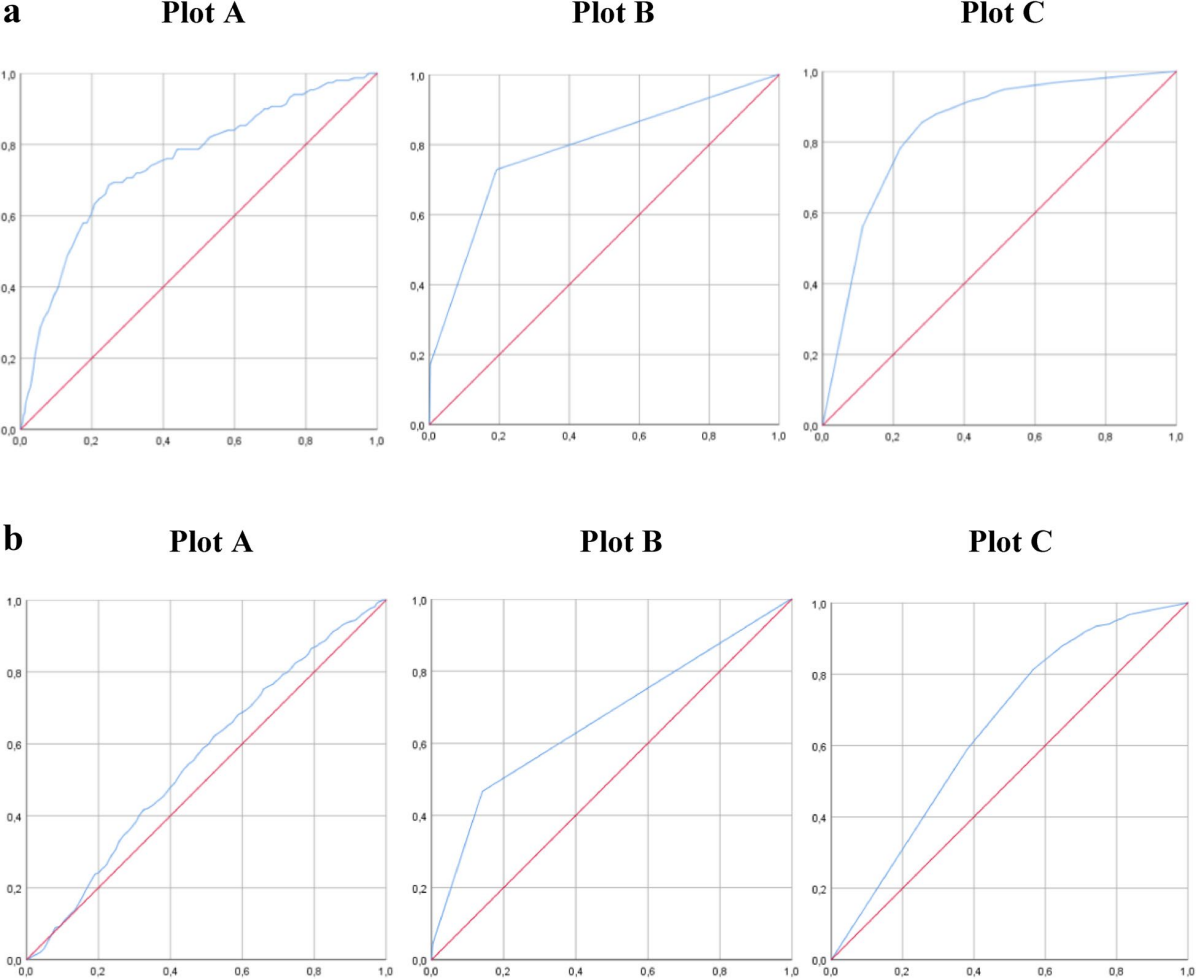
**a** Plot A represents the ROC AUC analysis for age as a discriminative variable for survival; B NACA score and survival; C represents prehospital GCS and survival. **b** Plot A represents the ROC AUC analysis for age as a discriminative variable for ISS; B NACA score and ISS > 16; C prehospital GCS and ISS > 16

The results of the ROC-AUC analysis are illustrated in Figs. 3 and described below. To quantify the effect of covariables on survival, the ROC area under the curve (AUC) analysis was made. The analysis showed that prehospital GCS, NACA score and age were good discriminant finding an AUC values of 0.840 (SE 0.019; 95% CI 0.802–0.877), of 0.784 (SE 0.022, 95% CI 0.741–0.828), and 0.750 (SE 0.022, 95% CI 0.707–0.793), respectively. However, when looking at the quantification of the effect of these covariables on the minimum ISS score of a polytrauma patient (≥ 16), the ROC AUC analysis identified a weak result in randomly guessing the predicting value of a polytrauma patient. The ROC AUC results were of 0.647 (SE 0.014; 95% CI 0.619–0.675) for prehospital GCS, 0.666 (SE 0.014; 95% CI 0.639–0.694) for NACA score, 0.557 (SE 0.014; 95% CI 0.531–0.584) for age. The identification of a very small number of the standard error indicated the high precision of the AUC analysis in all the aforementioned variables.

## Discussion

The main finding of this study is that NACA is a valid score for assessing polytrauma patients, correlating with both the pre-ER as well as the ER condition, as well as the post-hospitalization ISS assessment of patient severity.

These findings underline the ability of the NACA score in differentiating severity levels among polytraumatized patients, which is a key phase for health care professionals in properly managing these challenging patients. The correlation between higher NACA scores and increased mortality rates, as well as the necessity for intensive care confirmed its predictive power. In fact, a statistically significant difference was found at 28 days after discharge from the hospital between NACA 4 and NACA 5 patients. In NACA 4 patients there was a 97.7% survival rate during the hospital stay and among the survived individuals 3.5% died within 28 days after discharge, whereas in NACA 5 patients there was a 82.5% survival rate during the hospital stay and almost 23.5% of patients died within 28 days after discharge. The mortality rate stratified according to the NACA score aligns with earlier research on mortality [18]. Alongside, the analysis of the injury mechanisms revealed interesting patterns. Falls from less than 3 m were the predominant mechanism in NACA 4 patients, whereas road traffic accidents were more frequent in NACA 5 patients. This differentiation suggests that the NACA score reflects the nature and dynamics of the trauma, which could be important when providing specific medical procedures e.g. in the ER or in the operation room. To this regard, a statistical difference was found between NACA 4 and 5 both in the need for CPR and intubation.

The NACA score, which is performed pre-ER, also correlated with the ISS, a mandatory tool used worldwide for assessing trauma severity, based on the patient's injuries, in a post-hospitalization setting [4]. This study revealed a significant correlation between the NACA score and the ISS, highlighting that as the NACA score increased, the percentage of patients with an ISS greater than 16 also increased; e.g. 83.3% of patients with NACA 6 had an ISS over 16, compared to only 18.4% of those with NACA 4. This correlation demonstrates the complementary nature of the two scoring systems, with the NACA score providing immediate prehospital assessment consistently with ISS offering detailed scoring during and after hospital stay in a consistent way. Thus, while previous literature largely focused on characterizing patients with ISS, this study showed the potential of an earlier NACA assessment [19–21].

NACA also correlated with GCS, another important score largely used in the clinical practice to describe traumatic brain injuries and patient cognitive status. The neurological status of these patients plays an important role in predicting the outcome [15]. Gross et al. published a cohort study with 111 prospectively collected patients evaluating the functional outcome and quality of life in polytraumatized patients in a 2-year follow-up [22]. While, both groups experienced a significantly long-term outcome reduction in comparison with pre-injury level, the study identified a significantly higher working capacity decrease in polytraumatized patients with brain injury compared to non-traumatic brain injury polytraumatized patients [22]. Accordingly, the correlation of the severity of head trauma injury and the prehospital NACA clinical assessment is key for an early management of polytraumatized patients, and this study showed a significant correlation between GCS and NACA score, both in the prehospital and at the ER use.

A further analysis was performed to investigate the predictive value of the different identified variables. The ROC AUC analysis demonstrated a strong correlation between survival and variables such as the prehospital GCS, NACA, and age, which means that patients’ survival was predictable based on the severity of the neurological status, age, and the prehospital evaluation with the NACA score. However, when focusing the analysis on ISS > 16 patients, the ROC AUC analysis showed a minor role for age, while confirming the prehospital NACA score together with the GCS value as the strongest variables predicting the patients’ overall outcome and survival.

Despite its strengths, this study has some limitations. This study on 2239 patients provided a large number of NACA 4 and 5 patients, while other scores were less represented, thus impairing further subanalyses. Moreover, the retrospective nature of the study allowed only the analysis of the factors available in the registry, and future research should aim to refine the NACA score both by investigating accuracy and precision of this method, and by incorporating additional variables that could predict long-term outcomes more accurately. Finally, the NACA score is utilized not in all European countries, and a comparative analysis with other international trauma scoring systems could provide a more comprehensive understanding of its global applicability including larger datasets, such as the whole Swiss Trauma Registry as well as data from other countries. Overall, this study underlined the potential role of this score. By integrating the NACA score with other tools like the ISS and GCS, clinicians can have a more nuanced and accurate assessment of polytrauma severity, ultimately enhancing patient care in both prehospital and hospital settings. To this aim, the NACA score is capable in predicting patients’ outcome and mortality already from the earlies phase, which can help healthcare professional in the management of these delicate patients.

## Conclusions

This study provides valuable evidence supporting the effectiveness of the NACA score in assessing the severity of polytrauma patients. The NACA is a valid score for assessing polytrauma, especially in patients with score 4 or 5, correlating with both the pre-ER as well as the ER condition, as well as the post-hospitalization ISS assessment of patient severity. Future studies should confirm the results also for the other NACA grades.

## Data Availability

No datasets were generated or analysed during the current study.

## Abbreviations

NACA: National Advisory Committee for Aeronautics
ISS: Injury severity score
GCS: Glasgow Coma Scale
EMS: Emergency medical services
STR: Swiss Trauma Registry
ER: Emergency room
AIS: Abbreviated Injury Scale
CI: Confidence interval
SD: Standard deviation
CPR: Cardiopulmonary resuscitation
ROC: Receiver operating characteristic
AUC: Area under the curve
SE: Standard error
